# AI-supported estimation of safety critical wind shear-induced aircraft go-around events utilizing pilot reports

**DOI:** 10.1016/j.heliyon.2024.e28569

**Published:** 2024-03-21

**Authors:** Afaq Khattak, Jianping Zhang, Pak-Wai Chan, Feng Chen, Caroline Mongina Matara

**Affiliations:** a Key Laboratory of Infrastructure Durability and Operation Safety in Airfield of CAAC, College of Transportation Engineering, Tongji University, 4800 Cao'an Road, Jiading, Shanghai, 201804, China; b Second Research Institute of Civil Aviation Administration of China, Civil Unmanned Aircraft Traffic Management Key Laboratory of Sichuan Province, China; c Hong Kong Observatory, 134A Nathan Road, Kowloon, Hong Kong, China; d Department of Civil and Construction Engineering, University of Nairobi, P.O. Box 30197-00100, Nairobi, Kenya

**Keywords:** Aviation safety, Go-around, Wind shear, Machine learning, Data augmentation

## Abstract

The occurrence of wind shear and severe thunderstorms during the final approach phase contributes to nearly half of all aviation accidents. Pilots usually employ the go-around procedure in order to lower the likelihood of an unsafe landing. However, multiple factors influence the go-arounds induced by wind shear. In order to predict the wind shear-induced go-around, this study utilized a cutting-edge AI-based Combined Kernel and Tree Boosting (KTBoost) framework with various data augmentation strategies. First, the KTBoost model was trained, tested, and compared to other Machine Learning models using the data extracted from Hong Kong International Airport (HKIA)-based Pilot Reports for the years 2017–2021. The performance evaluation revealed that the KTBoost model with Synthetic Minority Oversampling Technique - Edited Nearest Neighbor (SMOTE-ENN)- augmented data demonstrated superior performance as measured by the F1-Score (94.37%) and G-Mean (94.87%). Subsequently, the SHapley Additive exPlanations (SHAP) approach was employed to elucidate the interpretation of the KTBoost model using data that had been treated with the SMOTE-ENN technique. According to the findings, flight type, wind shear magnitude, and approach runway contributed the most to the wind shear-induced go-around. Compared to international flights, Hong Kong-based airlines endured the highest number of wind shear-induced go-arounds. Shear due to the tailwind contributed more to the go-around than the headwinds. The runways with the most wind shear-induced Go-arounds were 07C and 07R.

## 1 Introduction

During the final approach phase of an aircraft, a considerable number of aviation accidents occur, primarily due to unfavorable weather conditions. To mitigate the risk of unsafe landings, the implementation of a go-around protocol is essential to preventing such incidents. Despite this established protocol to mitigate risky landings, the combination of intricate maneuvers and time constraints can exacerbate underlying risks, particularly when confronted with adverse weather conditions. In situations where go-arounds become necessary, they typically occur at lower speeds and altitudes. Therefore, it is crucial to respond swiftly and make appropriate adjustments to the aircraft's altitude, thrust level, and course to ensure safe maneuvering. The implementation of these steps serves the purpose of avoiding potential conflicts with air traffic in the surrounding area. Nevertheless, it is important to recognize that they can have adverse effects on airline punctuality and airport throughput and contribute to an increased workload for air traffic controllers and pilots [1,2].

**Fig. 1:**
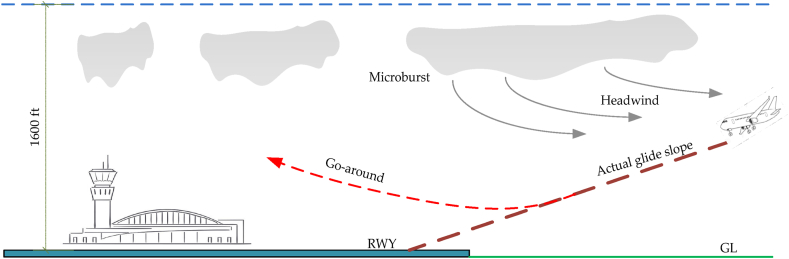
Wind shear occurrence location near airport runways.

Within the aviation sector, wind shear is a noteworthy meteorological event that demands careful attention. It is characterized by a consistent variation in wind speed, measuring 15 knots or higher, which can occur in either the opposite direction (headwind) or the same direction (tailwind) as the aircraft's forward motion. Wind shear has a tendency to impact the aerodynamic lift of an aircraft, which can cause deviations from the intended approach trajectory and have a detrimental effect on the aircraft's landing phase [3]. The implementation of the go-around protocol, as illustrated in Fig. 1, may ultimately take place.

The aspects and criteria that influence go-around occurrences have always been the subject of numerous studies. The efficiency and attitudes of pilots and air traffic controllers have been the key themes of many researchers assessments of go-around events. Pilots experience negative psychological effects associated with go-arounds, as the uncertainty surrounding the outcome can temporarily hinder their cognitive and decision-making abilities [4]. Another study revealed that pilots display unusual aerial behaviors during go-around maneuvers. These behaviors include deviations in flight paths and changes in visual scanning patterns [5]. Kennedy et al. [6] found that the experience and age of air traffic controllers significantly influence their decisions regarding go-arounds. In addition to the psychological aspects of pilots and air traffic controllers, several researchers have also examined the environmental factors that contribute to go-around occurrences. For instance, Zaal et al. [7] found that visibility, wind velocity, and localizer deviation significantly influence go-around decision-making. One study found that thunderstorms and winds exceeding 29 mph significantly increase the probability of initiating a go-around [8]. Another study revealed that convective storms along the landing glide slope are contributing factors to go-around situations [9].

In the field of aviation meteorology and safety, significant progress has been made in recent years in the adoption of artificial intelligence (AI), specifically machine learning techniques. These advancements have played a crucial role in enhancing various aspects of aviation operations, including weather forecasting, risk assessment, and safety management. The application of AI in this domain has allowed for more accurate predictions, improved decision-making processes, and ultimately, the enhancement of aviation safety. The rise in demand for advanced computational techniques to handle big data, such as those derived from Doppler Light Detection and Ranging (LiDAR) and Pilot Reports (PIREPs), is the main rationale behind this trend [10]. Recently, various researchers have used machine learning models to assess wind shear, aviation turbulence, flight delays and missed approaches such as Chou et al. [11] employed machine learning-based categorical boosting (CatBoost) approach to predict the aircraft missed approaches at Denver International Airport using various environmental aspects such as atmospheric pressure, visibility and speed of the wind. Similarly, a machine learning-based random forest (RF) model was proposed to estimate the flight delays at airports [12]. In another study, an online prediction of aircraft landing true airspeed and ground speed during the approach phase was carried our using RF model [13]. In these studies, machine learning models exhibited superior predictive performance; however, their “black-box” nature poses a substantial challenge in comprehending and interpreting the underlying mechanisms that drive their predictions [14]. This dearth of interpretability may pose a major concern. Comprehending the rationales underlying a model's decision (explainability) becomes a challenging task, thereby impeding confidence in the model for important purposes. There exist several interpretability techniques that efficiently address the challenges posed by black-box ML models and provide valuable insights into the decision-making process. These techniques include permutation-based feature importance analysis [15], Local Interpretable Model-agnostic Explanations (LIME) [16], and SHapley Additive exPlanations (SHAP) [17].

**Fig. 2:**
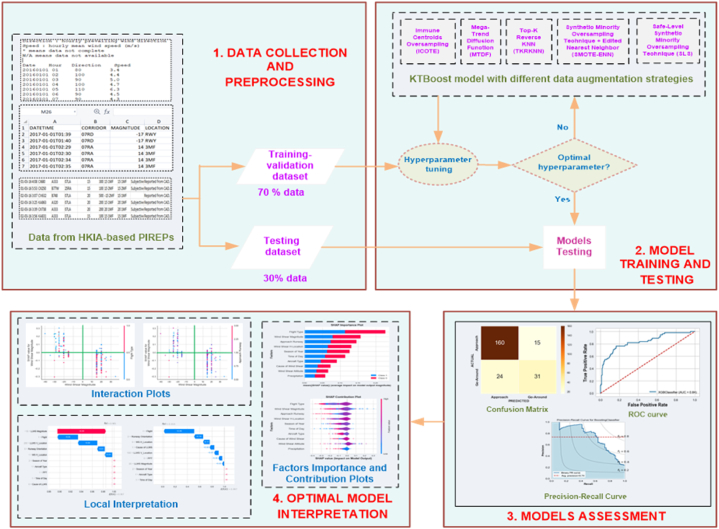
Proposed research framework for the prediction of wind shear-induced go-arounds.

To our knowledge, neither of these studies has provided an estimation of aircraft go-arounds specifically induced by wind shear events. Typically, aircraft go-around data is obtained from Pilot Reports (PIREPs), which tends to be skewed towards “landings/approaches” rather than go-around events. Therefore, the purpose of this study is to simultaneously deal with imbalance go-around data and estimate wind shear-induced go-arounds using a machine learning-based novel interpretable Combined Kernel and Boosting Tree (KTBoost) approach [18]. The reason to use the KTBoost approach is due to the fact that kernel boosting can generalize well to unseen data by using kernel functions to map data into a higher-dimensional feature space. Tree boosting, on the other hand, can handle local patterns and adapt to different regions of the feature space. By combining these two techniques, KTBoost can benefit from both global generalization and local adaptability, leading to improved overall generalization performance [19]. A broad range of environmental and situational factors that contribute to the go-around events were taken into account. The research utilized data obtained from PIREPs collected from Hong Kong International Airport (HKIA), which included several aspects such as altitude, magnitude, causes and encounter location of wind shear events, the aircraft type (narrow or wide-body) involved in a go-around, the weather conditions (clear sky or rainfall) during the go-around, the flight type (inbound international or domestic) at HKIA, the specific approach runway used for the go-around (07C, 07R, 07L, 25L, 25LC, or 25R), and temporal factors such wind shear-induced go around occurrence date and time. Similarly, in order to deal with data imbalance issue, several data balancing strategies were employed including Immune Centroids Oversampling (ICOTE) [20], Mega-Trend Diffusion Function (MTDF) [21], Top-K Reverse KNN (TKRKNN) [22], Synthetic Minority Oversampling Technique - Edited Nearest Neighbor (SMOTE-ENN) [23], and Safe-Level Synthetic Minority Oversampling Technique (SLS) [24]. In addition, post-hoc SHAP interpretation mechanism was employed to explain the KTBoost model and assess different contributing factors. Understanding the factors that contribute to go-around events can provide valuable insights to aviation policymakers. By analyzing these factors, policymakers can develop effective strategies to minimize the occurrence of go-arounds and enhance aviation safety. Fig. 2 illustrate the whole research framework.

The subsequent section of the paper is structured as follows.

● Section 2 provides details on the data source, an overview of the KTBoost model, and the SHAP method.
● In Section 3, a comparative analysis of the performance of the KTBoost model is conducted, along with other advanced machine learning models. Post-hoc interpretation outcomes using the SHAP method are also presented.
● The concluding section 4 summarizes the findings of the study.

## 2 Data and methods

### 2.1 Study location

**Fig. 3:**
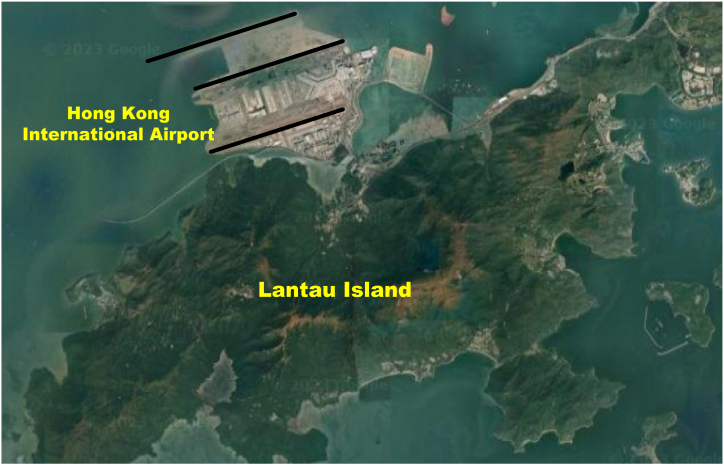
Complex terrain to the south of HKIA.

The Hong Kong International Airport (HKIA), identified by its IATA code HKG and ICAO code VHHH, serves as the primary aviation hub in Hong Kong. Situated on the man-made island of Lantau, it lies along the sub-tropical coastline of mainland China (Fig. 3). The typical convective weather patterns in Hong Kong encompass tropical cyclones and the southwest monsoon [25]. The presence of convective weather phenomena not only leads to flight delays at HKIA but also increases the likelihood of thunderstorms and heavy rainfall in this region. Due to this, HKIA exhibits a high vulnerability to wind shear events compared to other airports [26]. Such weather patterns can have significant impacts on aviation operations, posing challenges for pilots, air traffic controllers, and airport authorities. It is essential to closely monitor and assess convective weather conditions to ensure the safety and efficiency of flights in the region. Multiple studies, encompassing observational and simulation-based research, have consistently shown that Lantau Island to the south of HKIA has a distinct contrast between land and sea, along with complex terrain. In addition to severe weather conditions, the complex terrain is also an ideal condition to generate wind shear events in the airport vicinity [27].

### 2.2 Data processing

A PIREP short for Pilot Report is a report that provides information about the real-time weather conditions experienced by an aircraft during its flight [28]. Typically, such reports are transmitted via radio to a suitable ground station for distribution. Upon encountering weather conditions that may pose a potential hazard, pilots promptly inform air traffic controllers via PIREP. It typically contains the information of turbulence, icing, and the current state of the flight trajectory. Based on its vulnerability of HKIA to wind shear events, HKIA-based PIREPs include specific information regarding its frequency and characteristics. In addition, it includes the details regarding aircraft type and flight involved, the altitude above runway level at which a wind shear event is encountered (e.g., 50 ft, 150 ft, 500 ft), the wind shear encounter locations from the runway threshold (e.g., 1 MF, 2 MF, 3 MF), the magnitude and intensity of wind shear events as well as its date and time of occurrence. Pilots may also report go-around events in the HKIA-based PIREPs as given in Table 1.

**Table 1:** Parameters and sample data extracted from HKIA-based PIREPs.

**Date**	**Time**	**Approach Runway**	**Flight Type**	**Aircraft Type**	**Wind Shear Magnitude**	**Wind Shear Encounter Location**	**Wind Shear Altitude**	**PPT**	**Cause of Wind Shear**	**Go-around**
2018-09-12	3:46	07L	CX616	A333	−25 knots	1 MF	350 ft	Yes	See breeze	Yes
2019-03-29	17:35	25C	KA451	A333	20 knots	1 MF	200 ft	No	Gust Front	Yes
–	–	–	–	–	–	–	–	–	–	–
–	–	–	–	–	–	–	–	–	–	–
2020-08-17	8:38	07L	HX337	A333	31 knots	RWY	50 ft	No	Gust front	Yes
–	–	–	–	–	–	–	–	–	–	–
–	–	–	–	–	–	–	–	–	–	–
2020-11-07	21:43	25L	CX445	A359	−15 knots	2 MF	1000 ft	No	Sea breeze	Yes

NOTE: **PPT:** Precipitation.

The scope of this research is limited to go-arounds triggered by wind shear events. Therefore, in this study, we exclusively utilized information from incoming flights while excluding wind shear event data reported by outbound flights. Moreover, a Binary Classification Problem (BCP) was established by assigning the label “1″ to all instances of go-around (the minority class) and the label “0″ to all instances of approaches/landing (the majority class). Table 2 presents a comprehensive compilation of the variables, accompanied by detailed descriptions of each factor.

**Table 2:** Description of parameters from HKIA-based PIREPs with their label coding.

**Factors**	**Descriptions of Factors**	**Data Type**	**Label coding of categories of factors**
**Go-around**	Aircraft missed an approach due to wind shear	Discrete	1:The go-around protocol is executed during wind shear event. 0: Landing.
**Vehicle Specific**	Flight Type	Discrete	1: When go-around is carried out by an International inbound flight to HKIA. 0: In case of HK-based inbound flights.
	Aircraft Type	Discrete	1: The go-around is initiated by wide-body aircraft in the wind shear event. 0: In case of narrow-body aircraft during wind shear event.
**Runway Specific**	Assigned Runway for Landing	Discrete	1: In case when the designated approach runway is 07R and go-around is initiated. 2: In case when the designated approach runway is 25C and go-around is initiated. 3: In case when the designated approach runway is 25L and go-around is initiated. 0: In case when the designated approach runway is 07C and go-around is initiated.
**Environment Specific**	Wind shear Magnitude	Continuous	–
	Wind Shear H-Location	Discrete	1: In case when go-around is initiated at 1 nautical miles away from the runway threshold 2: In case when go-around is initiated at 2 nautical miles away from the runway threshold 3: In case when go-around is initiated at 3 nautical miles away from the runway threshold 0: In case when go-around is initiated the threshold of the runway
	Wind shear Altitude	Continuous	–
	Cause of wind shear	Discrete	In scenario 1, wind shear is caused by sea breeze, while in scenario 0, wind shear is caused by gust front.
	Precipitation	Discrete	1: if there is precipitation during go-around, 0: otherwise
**Temporal Specific**	Time of the Day	Discrete	Value of 1 is assigned to instances of go-around that occur between the hours of 08:00 and 18:59, indicating daytime. Conversely, a value of 0 is assigned to instances of go-around that occur between the hours of 19:00 and 07:59, indicating nighttime.
	Seasons of the Year	Discrete	1: In the event that a go-around occurs during the spring season, 2: In the event that a go-around occurs during the summer season, 3: In the event that a go-around occurs during the autumn season, 0: In the event that a go-around occurs during the winter season.

### 2.3 Theoretical overview of KTBoost model

Boosting algorithms are commonly employed in machine learning as well as data science research due to their exceptional predictive accuracy on complicated datasets. They iteratively combine weak classifiers to reduce both bias and variance. In several instances, learners commonly rely on just one kind of function as their base, despite their extensive application. In contrast, the KTBoost algorithm incorporates either a regression tree or a penalized reproducing kernel Hilbert space (RKHS) [29] into the ensemble of base classifiers during each iteration of boosting. In each boosting iteration (q), a candidate tree $\left[f_{q}^{T}\left(\chi \right)\right]$ and RKHS function $\left[f_{q}^{K}\left(\chi \right)\right]$ are utilized as the minimizers of the second order Taylor approximation $R^{2}\left(\Psi_{q}+f\right)$ using Newton or gradient optimization strategy. Subsequently, the base learner with the lowest empirical risk (R) is picked for inclusion in the ensemble. The KTBoost then selects either the tree or the RKHS function such that the addition of the base learner to the ensemble according to Eq. (1) results in the lowest risk.

(1) $$\psi_{q}\left(\chi \right)=\psi_{q-1}\left(\chi \right)+\nu \times f_{q}\left(\chi \right)$$

where, ν is the shrinkage factors, which is usually incorporated to update the equation. Furthermore, this amalgamation enables improved comprehension of functions with varying degrees of regularity, including both discontinuous and smooth portions. Discontinuities are typically learned by means of regression trees, while continuous or smooth portions are learned via RKHS functions. Following is a representation of the KTBoost algorithm in the form of pseudo code.

**Table:** 

**ALGORITHM:** KTBoost	
1	**Initialization:**$\Psi_{0}\left(\chi \right)={\mathrm{argmin}}_{C\in R^{d}}R\left(c\right)$
2	**for**q=1toQ**do**
3	Compute the functional gradient as $g_{q,i}=\frac{\partial }{\partial F}L\left(y_{i},\Psi \right){|}_{\Psi =\Psi_{q-1}\left(\chi_{i}\right)}$ and Hessian as $h_{q,i}=\frac{\partial^{2}}{\partial \Psi^{2}}L\left(y_{i},\Psi \right){|}_{\Psi =\Psi_{q-1}\left(\chi_{i}\right)}$ at the function $\Psi_{q-1}\left(\chi \right)$ and $I_{\left\{\chi =\chi_{i}\right\}}\left(\chi \right)$, where $I_{\left\{\chi =\chi_{i}\right\}}\left(\chi \right)=1$ if $\chi =\chi_{i}$, otherwise 0.
4	Determine the candidate regression tree and RKHS function as: $f_{q}^{T}\left(\chi \right)={\mathrm{argmin}}_{f\in \tau }R^{2}\left(\Psi_{q-1}+f\right)$ $f_{q}^{K}\left(\chi \right)={\mathrm{argmin}}_{f\in Η}R^{2}\left(\Psi_{q-1}+f\right)+\frac{1}{2}\lambda \times {\left\|f\right\|}_{Η}^{2}$ where $R^{2}\left(\Psi_{q-1}+f\right)$ is the approximate or empirical risk, which can be describe by expression: $R^{2}\left(\Psi_{q-1}+f\right)=\sum_{i=1}^{n}g_{q,i}\times f\left(\chi_{i}\right)+\frac{1}{2}h_{q,i}\times f{\left(\chi_{i}\right)}^{2}$ is
5	**if**$R^{2}\left(\Psi_{q-1}+v\times f_{q}^{T}\left(\chi \right)\right)\leq R^{2}\left(\Psi_{q-1}+v\times f_{q}^{K}\left(\chi \right)\right)$**then**
6	$f_{q}\left(\chi \right)=f_{q}^{T}\left(\chi \right)$
7	**else**
8	$f_{q}\left(\chi \right)=f_{q}^{K}\left(\chi \right)$
9	**End if**
10	Update $\Psi_{q}\left(\chi \right)=\Psi_{q-1}\left(\chi \right)+v\times f_{q}\left(\chi \right)$
11	**End for**

### 2.4 Performance metrics

The confusion matrix provides the basis for the metrics that are utilized in the evaluation of a BCP. These metrics include true positives $\left(\Delta_{p}\right)$, false positives $\left(\nabla_{p}\right)$, true negatives $\left(\Delta_{n}\right)$, and false negatives $\left(\nabla_{n}\right)$. When classifying the various elements that make up a BCP, the terms positive and negative are frequently utilized. When both the observed class and the predicted class are positive, this is referred to as a true positive. A situation is considered to have a false positive when it is predicted that the class will be positive when in reality, the class will be negative. To evaluate the efficacy of models, the following metrics in Eqs. (2), (3), (4), (5) are employed.

(2) $$Precision=\frac{\Delta_{p}}{\Delta_{p}+\nabla_{p}}\text{,}$$

(3) $$Recall=\frac{\Delta_{p}}{\Delta_{p}+\nabla_{n}}\text{,}$$

(4) $$F1-Score=\frac{\Delta_{p}}{\Delta_{p}+\frac{1}{2}\left(\nabla_{p}+\nabla_{n}\right)}\text{,}$$

(5) $$G-Mean=\sqrt{\left(\frac{\Delta_{p}}{\Delta_{p}+\nabla_{n}}\right)\left(\frac{\Delta_{n}}{\nabla_{p}+\Delta_{n}}\right)}\text{,}$$

#### 2.4.1 Precision-recall curve

In addition to metrics derived from confusion matrix, area under precision-recall curve (AU-PRC) is used to evaluate a classification model's quality, particularly when there is a problem with an unbalanced classification. When selecting and optimizing machine learning models, it is common to prioritize maximizing both recall and precision. Precision and recall are typically both between 0 and 1. As a result, a Precision-Recall curve with a sizable area underneath the curve would appear visually.

#### 2.4.2 Receiver operating characteristic (ROC) curve

The ROC curve is another performance metric for BCP at various threshold settings. Area under the Receiver Operating Characteristic Curve (AU-ROC) depicts the level or measure of separability. It shows how effectively the model can differentiate between classes. The greater the value of AU-ROC, the more accurately the model classifies negative classes as negative and positive classes as positive.

### 2.5 Model interpretation

The SHAP approach is a post-hoc explanation tool for the machine learning models. The fundamental principle that underlies the interpretation provided by the SHAP approach involves the computation of the individual contribution made by each factor towards the outcome of the model. This approach aims to explain and interpret the findings from a broader, global context as well as in a more specific, local context. During the process of training a model, the predicted values are determined for each individual instance. The Shapley value, also known as SHAP value is the assigned value for each factor within the given instance. Eq. (6) can be utilized to calculate the Shapley value, which represents the individual contribution of each factor.

(6) $$\varphi_{i}=\sum_{\mu \subseteq \Pi \left\{i\right\}}\frac{\mu !\left(n\;-\;\left|\mu \right|\;-\;1\right)!}{n!}\left[\hbar \left(\mu \cup \left\{i\right\}\right)\;-\;\hbar \left(\mu \right)\right]$$

where,

$\varphi_{i}$ Involvement of an $i^{th}$ input parameter from the dataset;

Π Subset of input parameter from the actual PIREPs dataset;

μ Subset of predicted parameters from the actual PIREPs dataset;

$\hbar \left(\mu_{i}\right)$ and ℏ(μ) Results obtained with and without inclusion of $i^{th}$ factor from the dataset.

The application of the SHAP approach utilizes an additive factors imputation strategy to generate an explainable machine learning strategy. The result of the algorithm is expressed as a linear combination and summation of all the input features, as demonstrated in Eq. (7).

(7) $$g\left(z^{\prime }\right)=\varphi_{0}+\sum_{i=1}^{\Lambda }\varphi_{i}z^{\prime }\text{,}$$

where,

$z^{\prime }\in {\left\{0,1\right\}}^{\Lambda }$ When the parameters is taken into account $z^{\prime }=1$, else $z^{\prime }=0$;

Λ Number of input parameters taken into consideration;

$\varphi_{0}$ Base value.

## 3 Results and discussion

In this study, the KTBoost model was used along with multiple data augmentation techniques to estimate the aircraft go-around induced by wind shear events. The analysis involved the assessment of PIREPs obtained from HKIA between 2017 and 2021. By analyzing both outbound and inbound PIREPs, a total of 1731 instances of wind shear were identified. Among these occurrences, 1388 instances were associated with inbound flights at HKIA, while 343 instances were linked to outbound flights. Table 3 provides detailed statistical information for each aspect covered in the HKIA-based PIREPs.

**Table 3:** Detailed statistical information of various aspects in HKIA-based PIREPs.

**Aspects**	**Factors**	**Descriptive statistics**			
		**Mean**	**Standard Deviation**	**Min**	**Max**
Runway Aspect	Assigned Approach Runway	0.900	1.002	0	3
Vehicle Aspect	Type of Flight	0.561	0.506	0	1
	Type of Aircraft Involved in Go-Around	0.739	0.428	0	1
Environmental Aspects	Wind Shear Altitude (ft)	336	305	15	2000
	Wind Shear Causes	0.461	0.489	0	1
	Precipitation (PPT)	0.541	0.486	0	1
	Wind Shear Magnitude (−/+)	−19.23/+17.17	4.85/3.86	−15/15	−40/45
	Wind Shear H-Location (nautical miles)	1.473	0.896	0	3
Temporal Aspects	Time of the Day	0.757	0.054	0	1
	Seasons of the Year	1.560	0.833	0	3

To begin the modeling process, the PIREPs dataset was divided into two separate sets: a training set and an evaluation set. The KTBoost model and other competitive machine learning models, including Extreme Gradient Boosting (XGBoost) [30], Adaptive Boosting (AdaBoost) [31], Explainable Boosting Machine (EBM) [32], Light Gradient Boosting Machine (LightGBM) [33], and a statistical Binary Logistic Regression (BLR) [34], were trained using the training dataset, which accounted for 70% of the total data. The remaining 30% of the dataset was used to assess the performance of these models.

### 3.1 Hyperparameter tuning via Bayesian optimization

Hyperparameter tuning is a critical step in optimizing the performance of the KTBoost model and other machine learning models. By fine-tuning the hyperparameters, it significantly improves the model's ability to generalize and reduces the risk of overfitting, ensuring more robust and accurate predictions. We employed Bayesian optimization technique [35] to identify the optimal hyperparameters for the KTBoost algorithm and other machine learning models. This technique combines surrogate models including Gaussian Processes or Tree-structured Parzen Estimators and an acquisition function (Expected Improvement, Probability of Improvement) to efficiently search the hyperparameter space and find the optimal set of hyperparameters [36]. The objective function is defined that quantifies the performance of machine learning model based on the chosen evaluation metric such as, accuracy, F1 score, G-Mean, etc. In this study, the G-Mean was employed as objective function, which was maximized in order to obtained the optimal hyperparameters. Two important hyperparameters considered for the KTBoost model were *learning_rate* and *n_estimators*. The *learning_rate* determines the step size at each boosting iteration, while *n_estimators* determines the number of boosting stages or iterations. These hyperparameters are crucial in controlling the model's learning rate and the overall complexity of the model, respectively. Tuning these hyperparameters can have a significant impact on the performance and generalization ability of the KTBoost model. Table 4 presents a comprehensive list of hyperparameters for KTBoost and other machine learning models. It comprises the hyperparameter search space and its associated optimal values.

**Table 4:** Optimal hyperparameters of KTBoost and other machine learning models.

**Models**	**Hyperparameters**	**Range**	**Optimal values**
KTBoost	*learning_rate*	(0.01–0.5)	0.08
	*n_estimators*	(50–2000)	550
LightGBM	*learning_rate*	(0.01–0.5)	0.07
	*reg_lambda*	(1–2)	1.24
	*n_estimators*	(50–2000)	900
AdaBoost	*n_estimators*	(50–2000)	750
	*max_depth*	(3–20)	8
XGBoost	*n_estimators*	(50–2000)	500
	*num_leaves*	(10–100)	60
	*learning_rate*	(0.01–0.5)	0.21
EBM	*n_estimators*	(50–2000)	620
	*learning_rate*	(0.01–0.5)	0.09

### 3.2 Comparison of KTBoost model with other machine learning models using original data

**Fig. 4:**
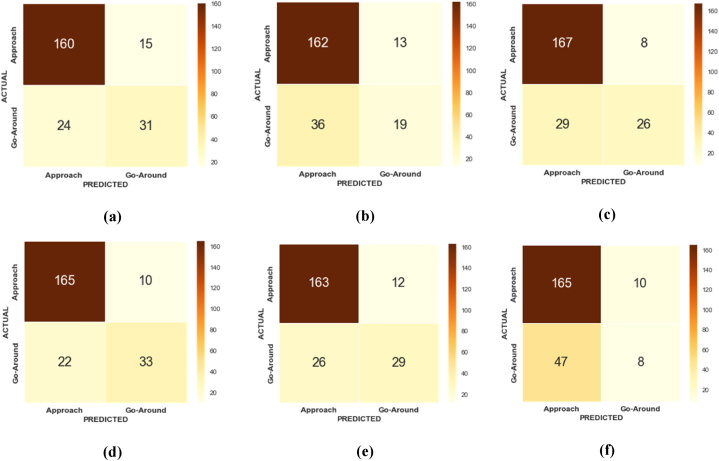
Confusion matrix for the machine learning models using original data; (a) XGBoost; (b) AdaBoost; (c) EBM; (d) KTBoost; (e) LightGBM; (f) BLR.

In this study, the confusion matrices derived from the original data using various machine learning models namely XGBoost, AdaBoost, EBM, KTBoost, LightGBM, and BLR present an insightful comparison of each model's ability to classify two critical classes: Go-around (positive class) and Approach (negative class). With the Go-around class being the minority and our focus, the models' effectiveness in accurately identifying this category is crucial. XGBoost model demonstrated a robust balance between sensitivity and specificity. It showed commendable accuracy in classifying the Go-around cases, although with a slight tendency to misclassify some Approach instances (Fig. 4a). AdaBoost presented a slightly higher rate of false negatives compared to XGBoost. Its strength lay in identifying the majority class (Approach) but at the cost of slightly lower sensitivity for the Go-around class (Fig. 4b). EBM exhibiting a comparable performance to XGBoost, the EBM model balanced the trade-off between identifying both classes. It showed a promising ability to reduce false positives (Fig. 4c). KTBoost model leaned towards high specificity but with a marginal increase in false negatives. KTBoost was efficient in Approach classification (Fig. 4d). Similar to EBM in its balanced approach, LightGBM offered an effective classification of the Go-around class. However, it also presented a moderate number of false positives, indicating a slight compromise in specificity (Fig. 4e). BLR as a baseline model showed limitations in distinguishing the Go-around class, leading to a higher number of false negatives. While it efficiently classified the Approach class, its overall sensitivity to the Go-around class was lower compared to other models (Fig. 4f).

According to the testing dataset, the KTBoost model trained with the original data outperformed other models, achieving a higher F1-score of 86.44% and G-Mean value of 76.07%, as indicated in Table 5. However, it is worth noting that out of the 55 go-around instances, only 33 instances were correctly classified, which is relatively low. Among the other models, the second-best performer was the XGBoost model, with an F1-score of 83.89% and a G-Mean value of 71.41%. This model correctly classified 31 instances of Go-around out of the total 55 instances. On the other hand, the BLR model exhibited the poorest performance, with an F1-score of 70.05% and a G-Mean of 37.36%. Only 8 instances of Go-around were correctly classified out of the 55 instances.

**Table 5:** Performance assessment of different machine learning models.

**Model**	**Performance Measures**			
	**Precision (%)**	**Recall (%)**	**F1-Score (%)**	**Geometric Mean (%)**
XGBoost	83.16	83.16	83.89	71.41
AdaBoost	75.36	79.16	77.31	57.71
EBM	83.67	84.14	82.51	67.77
KTBoost	**85.35**	**86.21**	**86.44**	**76.07**
LightGBM	83.56	83.76	83.65	70.96
BLR	70.66	75.91	70.05	37.36

**Fig. 5:**
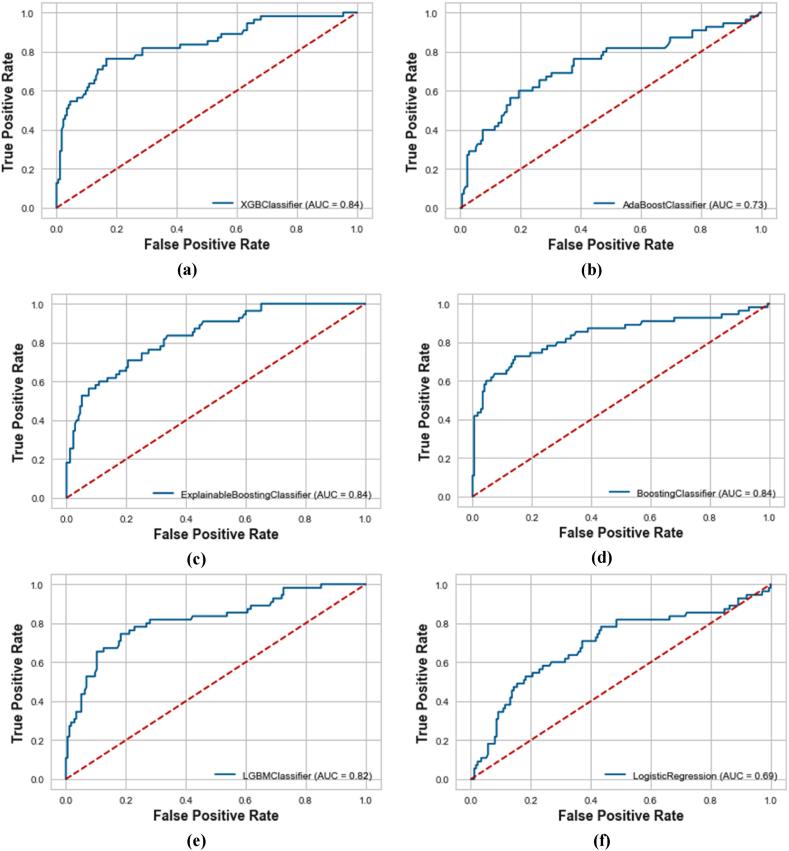
Receiver operating characteristic curves for the machine learning models using original data; (a) XGBoost; (b) AdaBoost; (c) EBM; (d) KTBoost; (e) LightGBM; (f) BLR.

**Fig. 6:**
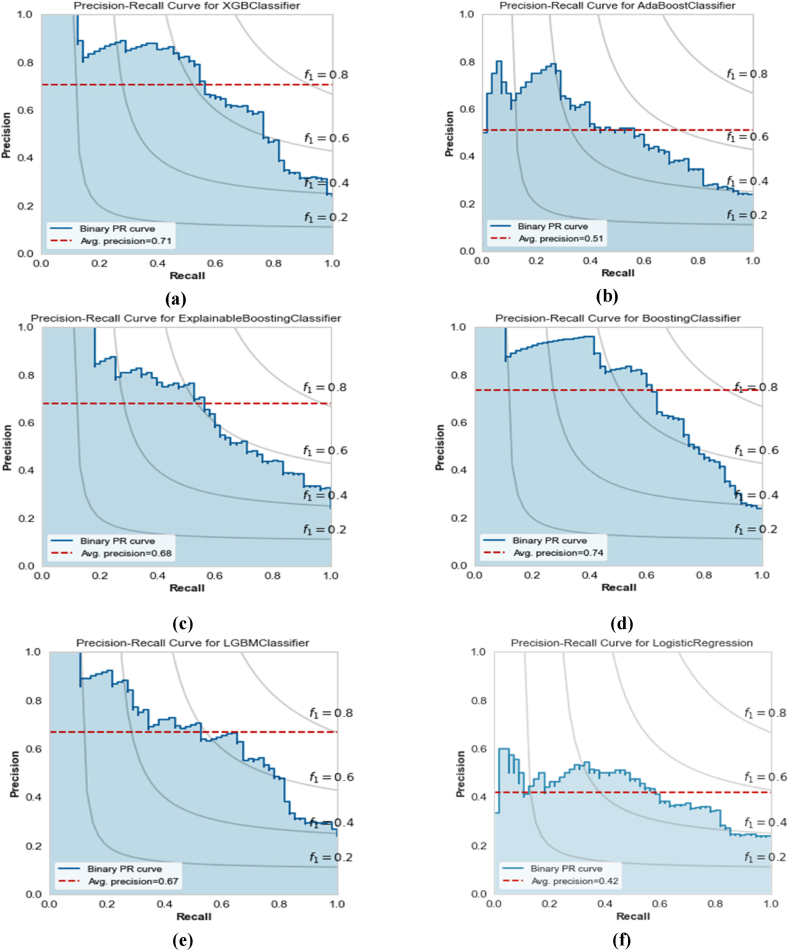
Precision-recall curves for the machine learning models using original data; (a) XGBoost; (b) AdaBoost; (c) EBM; (d) KTBoost; (e) LightGBM; (f) BLR.

The comparative analysis of the ROC curves and Precision-Recall Curves (PRC) for different machine learning models are shown in Fig. 5a-f and Fig. 6a-f, respectively. It has been revealed that XGBoost, EBM, and KTBoost achieved the highest AU-ROC value of 0.84, indicating a strong ability to discriminate between the positive and negative classes while BLR had the lowest AU-ROC of 0.69, illustrating less effectiveness in differentiating between the two classes. The KTBoost model stood out in the AU-PRC analysis with a value of 0.74, indicating superior performance in the precision-recall curve, which is particularly important in the context of imbalanced datasets where the Go-around class is a minority. Based on the original data, in case of overall comparison, the KTBoost model outperformed the other machine learning models in terms of overall performance metrics. Its balance between sensitivity (true positive rate) and precision makes it the ideal choice.

To further enhance the performance, various data augmentation strategies were employed to preprocess the input data for the KTBoost model, potentially leading to even better model performance.

### 3.3 Data treatment by various data augmentation strategies

**Fig. 7:**
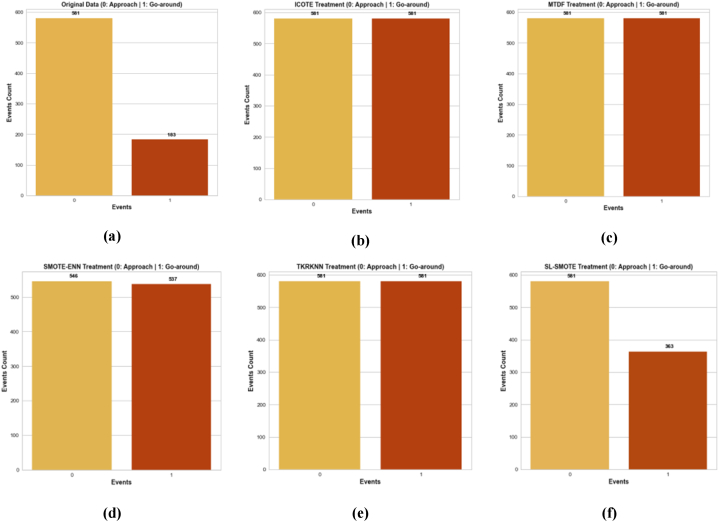
Data treatment by various data augmentation strategies; (a) Original data; (b) ICOTE treated data; (c) MTDF treated data; (d) SMOTE-ENN treated (e) TkRKNN treated data; (f) SL-SMOTE treated data.

The initial training dataset consisted of 581 instances of 'Approaches' and 183 instances of ‘wind-shear induced go-around’, resulting in an imbalance ratio of 0.31 as shown in Fig. 7a. Different data augmentation strategies were employed to balance the data. Fig. 7b-f illustrates the resampled training datasets after applying different data augmentation techniques.

● The ICOTE treatment produced a balanced dataset with 581 'approaches' records and 581 ′wind shear-induced go-around’ records, achieving a balancing ratio of 1 (Fig. 7b).
● The MTDF treatment also resulted in a balanced dataset with 581 'approaches' records and 581 ′wind shear-induced go-around’ records, with a balancing ratio of 1 (Fig. 7c).
● SMOTE-ENN treatment resulted in a balanced dataset with 546 'approaches' records and 537 ′wind shear-induced go-around’ records, with a balancing ratio of 0.98 (Fig. 7d).
● The TKRKNN treatment led to a balanced dataset with 581 'approaches' records and 581 ′wind shear-induced go-around’ records, achieving a balancing ratio of 1 (Fig. 7e).
● The SLS treatment resulted in a dataset with 581 'approaches' records and 363 ′wind shear-induced go-around’ records, with a balancing ratio of 0.624 (Fig. 7f).

### 3.4 Performance assessment of KTBoost model with treated data

**Fig. 8:**
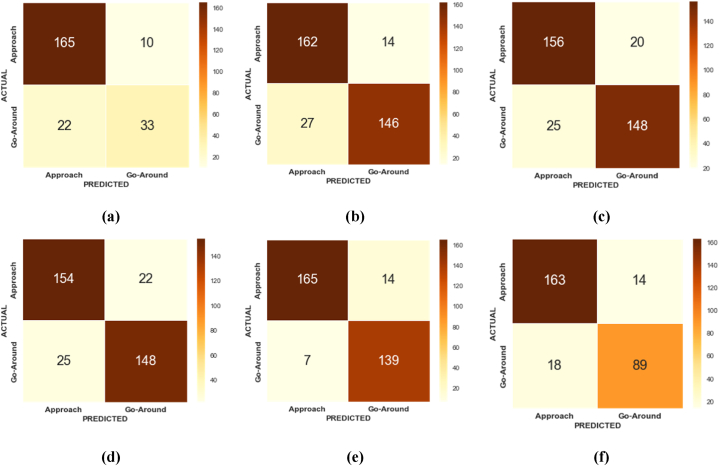
Confusion matrix of the KTBoost model with different treatment strategies; (a) KTBoost model with original data; (b) KTBoost model with ICOTE treated data; (c) KTBoost model with MTDF treated data; (d) KTBoost model with TKRKNN treated data; (e) KTBoost model with SMOTE-ENN treated data; (f) KTBoost model with SLS treated data.

In this study, to predict wind shear-induced go-arounds, state-of-the-art machine learning models were initially employed. However, these models under-performed for the minority class. The KTBoost model, optimized via Bayesian Optimization, was then chosen for its effectiveness in addressing this issue. Various treatment strategies were implemented with the KTBoost model to improve the probability of accurately classifying wind shear-induced go-arounds. Fig. 8 illustrates the confusion matrices for the KTBoost model under different data augmentation strategies, and the comparative analysis revealed that the KTBoost with Original Data (Fig. 8a) is baseline representation shows the initial performance of the KTBoost model without any data treatment. It provides a reference point to assess the impact of the subsequent data augmentation strategies. KTBoost with ICOTE Treated Data (Fig. 8b) shows an improvement in model performance. The confusion matrix indicates enhanced accuracy in predicting the minority class. KTBoost with MTDF Treated Data (Fig. 8c) further refines its ability to classify wind shear-induced go-arounds. The confusion matrix here likely shows a better balance between true positives and false negatives. KTBoost with TKRKNN Treated Data (Fig. 8d) display an optimization in the trade-off between sensitivity and specificity, evident from the changes in the confusion matrix. KTBoost with SMOTE-ENN Treated Data (Fig. 8e) results in significant improvements in the classification of the minority class, as reflected in its confusion matrix. KTBoost with SLS Treated Data (Fig. 8f) potentially showing a further enhancement in accurately predicting go-around instances. The corresponding performance indicators are extracted and presented in Table 6.

**Table 6:** Performance assessment KTBoost framework with data balanced via various strategies.

**Model with treatment strategy**	**Performance Measures**			
	**Precision (%)**	**Recall (%)**	**F1-Score (%)**	**Geometric Mean (%)**
KTBoost with original data	84.6	85.16	85.34	75.15
KTBoost with ICOTE treated data	88.34	88.83	88.54	88.36
KTBoost with MTDF treated data	87.57	87.15	87.32	87.61
KTBoost with TKRKNN treated data	86.15	86.45	86.16	86.21
KTBoost with SMOTE-ENN treated data	**94.35**	**94.73**	**94.37**	**94.87**
KTBoost with SLS treated data	90.25	90.11	90.34	90.15

Based on the testing dataset, the KTBoost model trained with SMOTE-ENN treated data outperformed other KTBoost models, achieving a higher F1-score of 94.37% and G-Mean value of 94.87%, as shown in Table 6. Out of the 146 instances of Go-around, 139 instances were correctly classified. The F1-score of the KTBoost model trained with SMOTE-ENN treated data was 9.17% higher than the KTBoost model trained with the original data. Similarly, the G-Mean value of the KTBoost model trained with SMOTE-ENN treated data was 24.71% higher than the KTBoost model trained with the original data. The worst performance was exhibited by the KTBoost model with TKRKNN treated data, with an F1-score of 86.16% and G-Mean value of 86.21%. However, these values were still better than those of the KTBoost model with the original data.

**Fig. 9:**
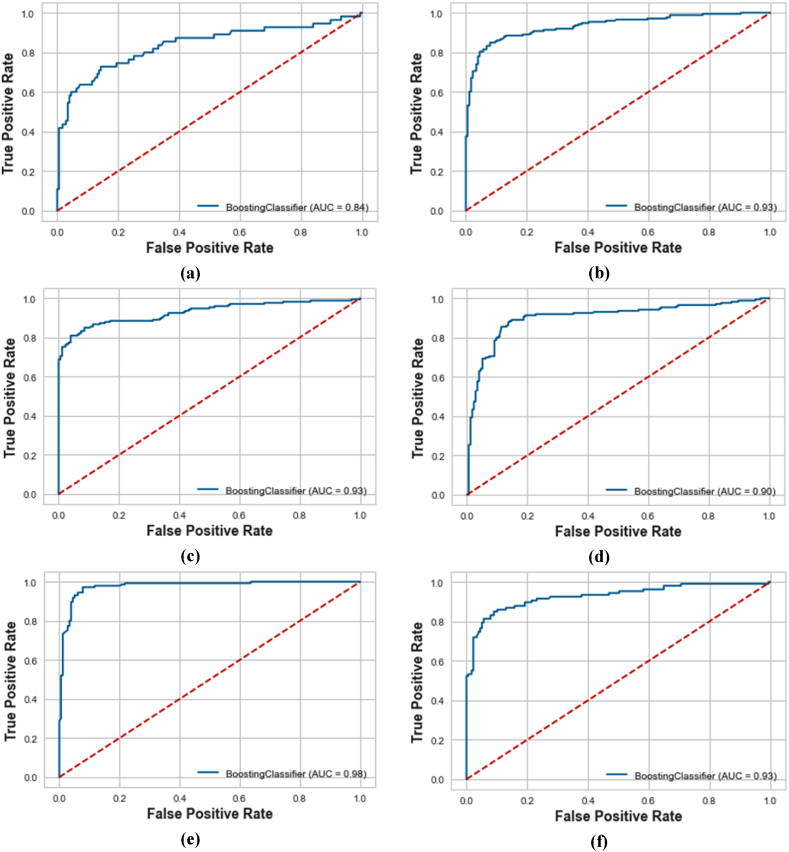
Receiver operating characteristic curves for the KTBoost model with different data augmentation strategies; (a) KTBoost model with original data; (b) KTBoost model with ICOTE treated data; (c) KTBoost model with MTDF treated data; (d) KTBoost model with TKRKNN treated data; (e) KTBoost model with SMOTE-ENN treated data; (f) KTBoost model with SLS treated data.

**Fig. 10:**
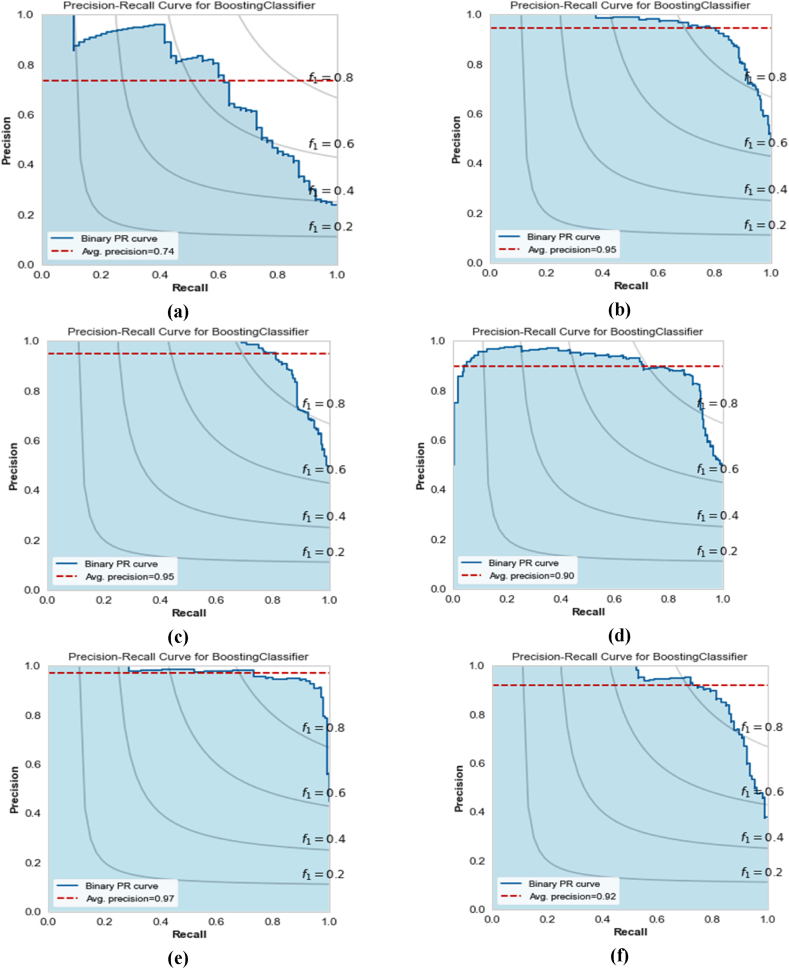
Precision-recall curves for the KTBoost model with different data augmentation strategies; (a) KTBoost model with original data; (b) KTBoost model with ICOTE treated data; (c) KTBoost model with MTDF treated data; (d) KTBoost model with TKRKNN treated data; (e) KTBoost model with SMOTE-ENN treated data; (f) KTBoost model with SLS treated data.

The ROC curves and PRC for the KTBoost model, applied with various data augmentation strategies, were examined. The corresponding AU-ROC and AU-PRC are depicted in Fig. 9, Fig. 10, respectively. The analysis of these metrics provides a clear understanding of the model's performance in accurately predicting wind shear-induced go-arounds. The KTBoost model with SMOTE-ENN treated data achieved the highest AU-ROC value of 0.98. This indicates an excellent ability of the model to distinguish between the go-around and approach classes. In terms of AU-PRC, again, the KTBoost model with SMOTE-ENN treated data outperformed the other models, achieving an AU-PRC value of 0.97. The superior performance of the KTBoost model with SMOTE-ENN treated data is evident in both AU-ROC and AU-PRC analyses. This demonstrates that the KTBoost model combined with SMOTE-ENN data treatment, is highly effective in correctly identifying wind shear-induced go-arounds.

### 3.5 SHAP-based interpretation of KTBoost model with SMOTE-ENN-treated data

Developing an accurate model for wind shear-induced go-arounds is indeed crucial, as it can provide valuable insights into the correlation between this event and various situational and environmental factors. The KTBoost model with SMOTE-ENN treated data has demonstrated optimal performance based on the performance measures. To further analyze the model and understand the impact of specific risk variables and their interactions, the SHAP approach was employed. This approach allows for a comprehensive assessment of the importance and influence of different features in predicting wind shear-induced go-arounds.

#### 3.5.1 Global Interpretation: importance and contribution of factors

**Fig. 11:**
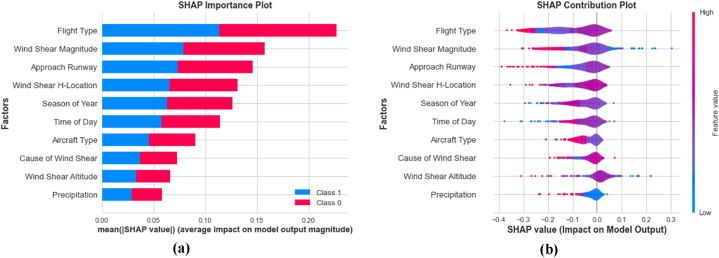
SHAP global interpretation; (a) SHAP importance plot (b) SHAP contribution plot.

It is crucial to distinguish between the concepts of “factor importance” and “factor contribution.” The importance of a given factor indicates the extent to which it contributes to the accuracy of a model. The outcomes can be logically explained by the factors observed through the analysis of factor contributions. Fig. 11a displays the SHAP-based global importance scores assigned to the factors in the KTBoost model using SMOTE-ENN-treated data. The primary factor contributing to the wind shear-induced go-around was the flight type, as indicated by a mean SHAP value of +0.23. Subsequently, wind shear magnitude exhibited a mean SHAP value of +0.160, while the approach runway had a value of +0.140.

Afterwards, a SHAP-based factor contribution analysis was conducted using SHAP bee-swarm plot (Fig. 11b). It provides a visual representation of the contributions of different factors to the predictions made by the KTBoost model. In the SHAP bee-swarm plot, the input factors are ranked in descending order of influence on the vertical axis. The most influential factor is placed at the top, while the least influential factor is at the bottom. The plot visually represents the impact of each factor using the SHAP value on the horizontal plane. The color scale ranges from blue (indicating low significance) to red (indicating high significance), providing an intuitive understanding of the relative importance of each factor in the model's predictions. The SHAP bees-warm plot of the KTBoost model with SMOTE-ENN treated data illustrated that HKIA-based flights endured most number of wind shear-induced go-around. The findings are derived from the analysis of PIREPs data spanning from 2017 to 2021, and one potential explanation for the results is the decreased frequency of international flights to Hong Kong due to the impact of the COVID-19 pandemic [37]. Another possible reason for the observed results could be the presence of numerous passenger and freight airlines at HKIA. Airlines such as Cathay Pacific, HK Express, Hong Kong Airlines, Air Hong Kong, Cathay Cargo, and Hong Kong Airlines Cargo operate from HKIA, making it a major hub for air transportation [38,39]. Similarly, it was observed that tailwind shear was associated with a higher frequency of initiated go-arounds compared to headwind shear. The tailwind shear phenomenon affects the lift of aircraft during the final approach, which could prevent an aircraft from touching down at the designated touchdown zone. These outcomes also aligns with previous studies [40,41]. The assigned approach runway was identified as the third most influential factor, with go-around initiation occurring more frequently on Runways 07C and 07R at HKIA. Previous studies have indicated that southerly or southeasterly gusts of wind at HKIA have a higher likelihood of causing wind shear, which can significantly impact runways 07C and 07R. These findings were derived from numerical simulations and wind tunnel testing [42,43]. Consequently, aircraft approaching these runways may experience strong wind shear, leading to the need for go-arounds. In conditions of high wind shear, it is not recommended to attempt landings on Runways 07C and 07R due to safety concerns associated with go-arounds.

#### 3.5.2 Factors interaction

**Fig. 12:**
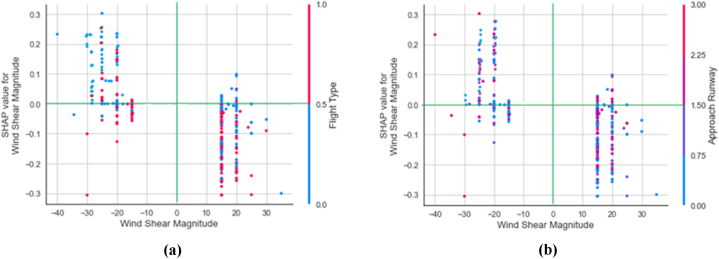
Interaction plots; (a) Wind shear magnitude and flight type interaction plot: (b) Wind shear magnitude and approach runway interaction plot.

The bee-swarm chart depicting the significance and contribution of factors showed no correlation between changes in factor value and variations in SHAP value. Fig. 12 provides supplementary information that complements the contribution plot. The analysis illustrated how different factors were interpreted by individuals and showed the variability of SHAP values in relation to eigenvalues. The SHAP-generated interaction plots were utilized to assess the influence of individual input factors on the final score obtained from the KTBoost model applied to SMOTE-ENN treated data.

Fig. 12a illustrates the effect of flight type and wind shear magnitude on model predictions. From −15 knots to −30 knots, points above the SHAP 0.00 green reference line have high densities. The vast bulk of the points have blue labels denoting HKIA-based domestic flights. It showed that domestic flights based at HKIA observed the majority of go-arounds during severe tailwind shear. Contrary to that, headwind shear and international flights were not the primary causes of the go-around. Furthermore, the relationship between wind shear magnitude and the approach runway is shown in Fig. 12b. It has been observed that most of the tailwind shear were occurred at Runway 07C and 07R. These findings are also consistent with the conclusions drawn from earlier studies [27,44,45].

#### 3.5.3 Local interpretation

**Fig. 13:**
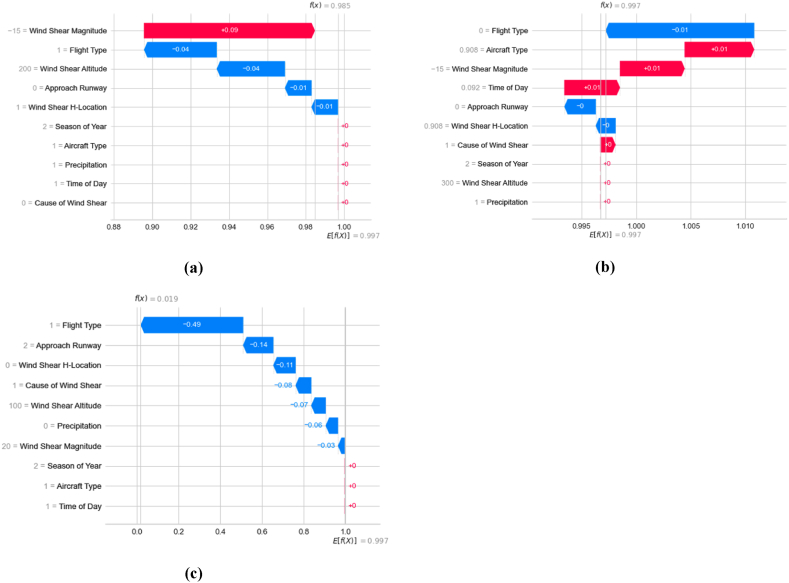
Local factor interpretation; (a) random instance of correctly classified ‘go-around’ with *f*(x) = 0.985, (b) random instance of correctly classified ‘go-around’ with *f*(x) = 0.997, (c) random instance of correctly classified ‘approach’ with *f*(x) = 0.019.

It is possible to provide an explanation for the KTBoost model's local estimation of the wind shear-induced go-around with the assistance of waterfall plots (Fig. 13). The waterfall plot displays each instance's contribution to the KTBoost prediction in descending order of magnitude; however, this order may not coincide with that of the overall results displayed in the bee-swarm plot. In each waterfall plot, the factor contribution to the prediction made by the KTBoost model is represented by blue and red bars with the associated magnitudes (blue denotes a negative value, and red denotes a positive value). The numbers that are displayed to the left of every factor in the waterfall chart are symbolic representations of the instance's factor values. The predictions made by the model are depicted as f(x) in the upper left corner of each waterfall plot, along with the actual values that correspond to them (obtained through PIREPs). Fig. 13a-c illustrate the KTBoost model predictions based on three randomly selected instances. Two of them are correctly classified as ‘Go-around’ and one as ‘Approach’. In point of fact, the bee-swarm plot demonstrated that the flight type factor was, all things considered, the most vital aspect in the process of estimating wind shear-induced go-around. However, in the case of the waterfall plot, a randomly selected instance that correctly classified as ‘Go-around’ showed that wind shear magnitude is the most significant factor, subsequently followed by the flight type aspect (Fig. 13a). The effect of flight type has been considered to be the most important in two of the three randomly selected instances (Fig. 13b and c), which has also been consistent with the bee-swarm plot. This can be seen by examining the data presented in these figures.

## 4 Conclusions and recommendations

In this research, we proposed a novel KTBoost framework with different data augmentation strategies for the estimation of wind shear-induced go-around events. In addition, SHAP analysis was used to interpret the model and identify the contributions of different factors. Wind shear-induced go-around events were estimated based on wind shear circumstances, taking into account environmental and situational factors. The analysis utilized wind shear data from PIREPS at HKIA between 2017 and 2021. The study yields the following conclusions.

● Initially, the KTBoost model was developed and compared to the other state-of-the-art models, including XGBoost, AdaBoost, EBM, LightGBM, and BLR, based on the original untreated training dataset from the HKIA-based PIREPs. The KTBoost model outperformed other models in terms of an F1-score of 86.44%, a G-Mean of 76.07%, an AU-ROC of 0.84, and an AU-PRC of 0.71.
● With an F1-score of 70.05% and a G-Mean of 37.36%, the statistical BLR model demonstrated the worst performance of all models based on the untreated data. The KTBoost F1-Score and G-Mean are 16.39% and 32.71% higher than the BLR model's F1-Score and G-Mean, respectively.
● Various data augmentation strategies were employed for the treatment of imbalanced PIREPs data and improving the performance of the KTBoost model. It was observed that the KTBoost model with the SMOTE-ENN treated data outperformed the others with an F1-Score of 94.37%, a G-Mean of 94.87%, an AU-ROC of 0.98, and an AU-PRC of 0.97.
● The SHAP approach proved successful in interpreting the optimal model's outcome (the KTBoost model with the SMOTE-ENN treated data). The SHAP finding demonstrates that the flight type, wind shear magnitude, and approach runway were the most significant contributors to wind shear-induced go-arounds.
● Most of the wind shear-induced go-arounds were observed by the HKIA-based domestic flights during the severe shear from tailwinds. Runways 07C and 07R were observed to be highly vulnerable to the occurrence of wind shear-induced go-arounds.

Recognizing the complex relationships between various risk factors that contribute to wind shear-induced go-arounds is crucial for aviation and meteorological applications. Therefore, researchers in contemporary civil aviation safety should seize this opportunity to gain insights in this field of research.

## Data availability statement

The data can be obtained from the corresponding author upon request.

## CRediT authorship contribution statement

**Afaq Khattak:** Writing – original draft, Software, Formal analysis, Conceptualization. **Jianping Zhang:** Project administration, Investigation, Funding acquisition. **Pak-Wai Chan:** Writing – review & editing, Supervision, Project administration, Funding acquisition. **Feng Chen:** Visualization, Supervision, Resources, Funding acquisition. **Caroline Mongina Matara:** Writing – review & editing, Visualization, Validation, Formal analysis.

## Declaration of competing interest

The authors declare the following financial interests/personal relationships which may be considered as potential competing interests: Afaq Khattak reports financial support was provided by 10.13039/501100001809National Natural Science Foundation of China. If there are other authors, they declare that they have no known competing financial interests or personal relationships that could have appeared to influence the work reported in this paper.

## References

[1] Jou R.-C., Kuo C.-W., Tang M.-L. (2013). A study of job stress and turnover tendency among air traffic controllers: the mediating effects of job satisfaction. Transport. Res. E Logist. Transport. Rev..

[2] Blajev T., Curtis W. (2017).

[3] Bretschneider L. (2021). Wind shear of low-level jets and their influence on manned and unmanned fixed-wing aircraft during landing approach. Atmosphere.

[4] Causse M. (2013). The effects of emotion on pilot decision-making: a neuroergonomic approach to aviation safety. Transport. Res. C Emerg. Technol..

[5] Dehais F. (2017). Pilot flying and pilot monitoring's aircraft state awareness during go-around execution in aviation: a behavioral and eye tracking study. Int. J. Aero. Psychol..

[6] Kennedy Q. (2010). Age and expertise effects in aviation decision making and flight control in a flight simulator. Aviat Space Environ. Med..

[7] Zaal P. (2019). AIAA Aviation 2019 Forum.

[8] Donavalli B., Mattingly S.P., Massidda A. (2017).

[9] Proud S.R. (2015). Analysis of aircraft flights near convective weather over Europe. Weather.

[10] Khattak A. (2023). Interpretable ensemble imbalance learning strategies for the risk assessment of severe‐low‐level wind shear based on LiDAR and PIREPs. Risk Anal..

[11] Chou C.-S., Tien A., Bateman H. (2021). 2021 IEEE/AIAA 40th Digital Avionics Systems Conference (DASC).

[12] Gui G. (2019). Flight delay prediction based on aviation big data and machine learning. IEEE Trans. Veh. Technol..

[13] Puranik T.G., Rodriguez N., Mavris D.N. (2020). Towards online prediction of safety-critical landing metrics in aviation using supervised machine learning. Transport. Res. C Emerg. Technol..

[14] Azodi C.B., Tang J., Shiu S.-H. (2020). Opening the black box: interpretable machine learning for geneticists. Trends Genet..

[15] Altmann A. (2010). Permutation importance: a corrected feature importance measure. Bioinformatics.

[16] Silva R.M. (2023). Developing and assessing a human-understandable metric for evaluating local interpretable model-agnostic explanations. Int. J. Intell. Eng. Syst..

[17] Lundberg S.M., Lee S.-I. (2017). A unified approach to interpreting model predictions. Adv. Neural Inf. Process. Syst..

[18] Sigrist F. (2021). KTBoost: combined kernel and tree boosting. Neural Process. Lett..

[19] Hussain S. (2022). Electric theft detection in advanced metering infrastructure using Jaya optimized combined Kernel‐Tree boosting classifier—a novel sequentially executed supervised machine learning approach. IET Gener. Transm. Distrib..

[20] Ai X. (2015). Advances in Knowledge Discovery and Data Mining: 19th Pacific-Asia Conference, PAKDD 2015, Ho Chi Minh City, Vietnam, May 19-22, 2015, Proceedings, Part I 19.

[21] Li D.-C. (2007). Using mega-trend-diffusion and artificial samples in small data set learning for early flexible manufacturing system scheduling knowledge. Comput. Oper. Res..

[22] Tsai M.-F., Yu S.-S. (2016). Distance metric based oversampling method for bioinformatics and performance evaluation. J. Med. Syst..

[23] Batista G.E., Prati R.C., Monard M.C. (2004). A study of the behavior of several methods for balancing machine learning training data. ACM SIGKDD Explor. Newsl..

[24] Bunkhumpornpat C., Sinapiromsaran K., Lursinsap C. (2009). Advances in Knowledge Discovery and Data Mining: 13th Pacific-Asia Conference, PAKDD 2009 Bangkok, Thailand, April 27-30, 2009 Proceedings 13.

[25] Lui G., Liem R., Hon K. (2020). IOP Conference Series: Earth and Environmental Science.

[26] Wong W.-K., Lau C.-S., Chan P.-W. (2013). Aviation model: a fine-scale numerical weather prediction system for aviation applications at the Hong Kong International Airport. Adv. Meteorol..

[27] Chan P., Hon K., Li Q. (2020). A comprehensive study of terrain‐disrupted airflow at Hong Kong International Airport–observations and numerical simulations. Weather.

[28] Kelsch M., Wharton L. (1996). Comparing PIREPs with NAWAU turbulence and icing forecasts: issues and results. Weather Forecast..

[29] Robert C. (2014).

[30] Chen T., Guestrin C. (2016). Proceedings of the 22nd ACM Sigkdd International Conference on Knowledge Discovery and Data Mining.

[31] Schapire R.E. (1999). Ijcai.

[32] Nori H. (2019).

[33] Ke G. (2017). Lightgbm: a highly efficient gradient boosting decision tree. Adv. Neural Inf. Process. Syst..

[34] King J.E. (2008). Binary logistic regression. Best practices in quantitative methods.

[35] Snoek J., Larochelle H., Adams R.P. (2012). Practical bayesian optimization of machine learning algorithms. Adv. Neural Inf. Process. Syst..

[36] Watanabe S. (2023). Tree-structured Parzen estimator: understanding its algorithm components and their roles for better empirical performance. arXiv preprint.

[37] Yuen A., Tsui K., Fung M. (2020). Aviation Law and Policy in Asia.

[38] Warnock-Smith D. (2021). Impact of COVID-19 on air transport passenger markets: examining evidence from the Chinese market. J. Air Transport. Manag..

[39] Kuo P.-F. (2022). The impact of the COVID-19 pandemic on OD flow and airport networks in the origin country and in Northeast Asia. J. Air Transport. Manag..

[40] Tse S., Chan P., Wong W. (2014). A case study of missed approach of aircraft due to tailwind associated with thunderstorms. Meteorol. Appl..

[41] Khattak A. (2022). Prediction of aircraft go-around during wind shear using the dynamic ensemble selection framework and pilot reports. Atmosphere.

[42] Tse L.K., Hon K., Li L.K. (2020). Large‐eddy simulations of neutrally stratified airflow over the complex terrain around Hong Kong International Airport with a three‐runway system. Meteorol. Appl..

[43] Louis K., Guan Y., Li L.K. (2021). RANS simulations of terrain-disrupted turbulent airflow at Hong Kong International Airport. Comput. Math. Appl..

[44] Hon K.K., Chan P.w. (2022). Historical analysis (2001–2019) of low‐level wind shear at the Hong Kong International Airport. Meteorol. Appl..

[45] Hon K., Chan P. (2014). Application of LIDAR‐derived eddy dissipation rate profiles in low‐level wind shear and turbulence alerts at Hong Kong International Airport. Meteorol. Appl..

